# You can't always get what you want: size assortative mating by mutual mate choice as a resolution of sexual conflict

**DOI:** 10.1186/1471-2148-9-129

**Published:** 2009-06-10

**Authors:** Sebastian A Baldauf, Harald Kullmann, Stefanie H Schroth, Timo Thünken, Theo CM Bakker

**Affiliations:** 1 Institute for Evolutionary Biology and Ecology, University of Bonn, An der Immenburg 1, D-53121 Bonn, Germany

## Abstract

**Background:**

Assortative mating patterns for mate quality traits like body size are often observed in nature. However, the underlying mechanisms that cause assortative mating patterns are less well known. Sexual selection is one important explanation for assortment, suggesting that i) one (usually the female) or both sexes could show preferences for mates of similar size or ii) mutual mate choice could resolve sexual conflict over quality traits into assortment. We tested these hypotheses experimentally in the socially monogamous cichlid fish *Pelvicachromis taeniatus*, in which mate choice is mutual.

**Results:**

In mate choice experiments, both sexes preferred large mates irrespective of own body size suggesting mating preferences are not size-assortative. Especially males were highly selective for large females, probably because female body size signals direct fitness benefits. However, when potential mates were able to interact and assess each other mutually they showed size-assortative mating patterns, i.e. the likelihood to mate was higher in pairs with low size differences between mates.

**Conclusion:**

Due to variation in body size, general preferences for large mating partners result in a sexual conflict: small, lower quality individuals who prefer themselves large partners are unacceptable for larger individuals. Relative size mismatches between mates translate into a lower likelihood to mate, suggesting that the threshold to accept mates depends on own body size. These results suggest that the underlying mechanism of assortment in *P. taeniatus *is mutual mate choice resolving the sexual conflict over mates, rather than preference for mates of similar size.

## Background

Sexual selection is a general selective force that has been intensively investigated in the past decades [see [1]]. The form and intensity of sexual selection are highly variable among species. In species in which mating is non-random, females are often expected to be choosy, especially when male parental investment is low. However, choosiness may also occur in males and should be promoted in both sexes when variances in quality of potential mates and parental investment are high [2,3]. Hence, mating preferences in each sex could generate a zone for sexual conflict, e.g. over mate quality [3,4].

Body size plays an important role in sexual selection of many species [1,5,6], including humans [7,8]. Usually, larger individuals are favored. For example, larger males may be preferred by females because of direct benefits, for instance better territorial defence [e.g. [9]], thereby providing better environmental conditions for raising offspring [10]. However, in the field and laboratory size-assortative mating is often reported and therefore much research has concentrated on its occurrence and the causal mechanisms [e.g. [11-14]]. Nevertheless, knowledge about assortment and its underlying mechanisms in mating systems with mutual mate choice is scarce.

Many mechanisms may lead to assortative mating patterns, for example spatial or temporal distribution. However, mate choice is one important explanation for assortment [see [15] and citations therein], and provides several different mechanisms that may result in assortative mating patterns. First, one sex (usually the female) or both sexes may show assortative mating preferences, e.g. with respect to body size [16,17]. Second, assortment may be the consequence of intra-sexual competition [18], so that only the larger winning individual is able to choose its mating partner. For example, in the curculionid beetle *Diaprepes abbreviatus *only competitive large males chose large, highly fecund females, whereas the losers had to accept the females of lower quality [19,20]. Third, mutual mate choice could explain assortment by resolving sexual conflict over mate quality traits [3,4]. When both sexes show preferences [21] for high quality mates, irrespective of own quality, low quality mates may be unacceptable. As a consequence, only high quality individuals would find partners which meet their preference. In contrast, low quality individuals would fail to mate at all [4], unless they show a choice threshold that accepts potential mates correlating to their own quality. In this case, resulting mating patterns would be assortative.

To test this hypothesis, it is necessary to show 1) directional mating preferences of both sexes, i.e. to exclude assortative preferences, and 2) size-assortative mating patterns independent of intra-sexual competition.

The study organism, *Pelvicachromis taeniatus*, is a cave-breeding cichlid that is characterized by a high level of cooperative biparental care [22]. Parental care often has high costs [2,3,23], e.g. due to predation risk, and time and energy loss. Thus, the probability of future mating may be negatively influenced by parental effort. When the costs of biparental care are high, males and females should be choosy [2-4]. Both sexes of *P. taeniatus *make a large investment in brood care and were choosy in prior experiments [22,24]. Pairs are socially monogamous and alternative mating tactics have not been observed yet. The males' testis size is very small, even suggesting genetic monogamy; however, the sperm length is extraordinarily long [25]. Males and females show sexual dimorphism in body size. Males are usually larger than females. Furthermore, both sexes are conspicuously colored. Males show, among others, a yellow nuptial coloration of their ventral body, while females develop a violet ventral coloration. During courtship, both sexes present their ventral region by arching it towards the partner while intensely quivering the whole body. After mutual mate choice and spawning the female cares for the eggs in the cave while the male defends the territory against intruders. The fry swims free after about one week and is then guarded by both parents.

The aim of our study was to test (1) whether mating preferences for body size of males and females are present in *P. taeniatus *and (2) the impact of size differences between potential mates on the likelihood to mate when partners are able to interact and assess each other mutually. In order to measure mating preferences for body size, we conducted a series of choice experiments using computer animations of digital images of different sizes of the opposite sex as stimuli. Test fish that greatly varied in body size were chosen for the experiments, thus preferences for assortative mating could be tested. A general problem when investigating preferences for body size is that size is often correlated with other factors. For example, large individuals might differ in behavior from smaller ones. A striking advantage of computer-manipulated stimuli is a high degree of standardization between the stimuli [26,27], thus minimizing the effects of confounding variables like rapid changes in coloration or different responses in stimuli fish.

In a second experiment, the consequences of size difference between the sexes on the likelihood to mate as well as the brood care intensity after successful mating were tested. Furthermore, we investigated whether body size could signal potential mate quality by analyzing variables such as the amount of parental investment, egg number of females and the number of surviving offspring.

## Results

### Size preferences in computer animations

#### Male mate choice

**Figure 1:**
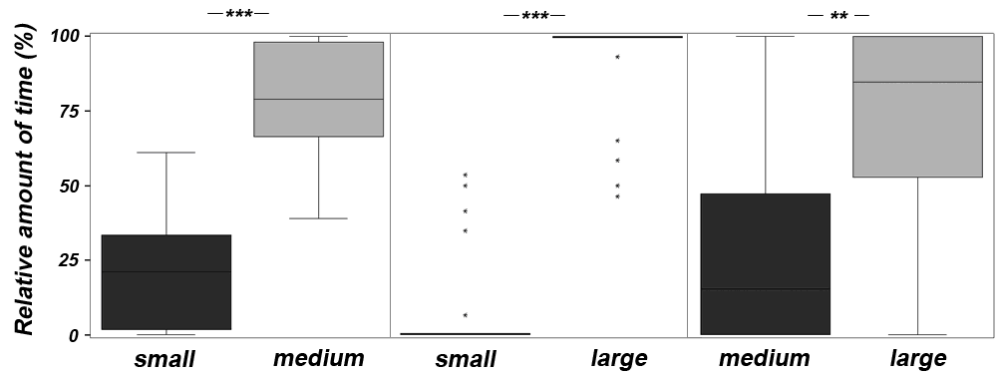
**Male preference**. Male preference expressed as percent of time spent in the association zone in front of female stimuli of different body size. Plotted are median, quartiles, percentiles. *** p < 0.001, ** p < 0.01, * p < 0.05, n.s. = not significant.

Males showed a highly significant preference for the larger female stimulus in each treatment (small vs. medium: n = 19, z = 3.601, p < 0.001; small vs. large: z = -4.087, n = 21, p < 0.001; medium vs. large: n = 24, z = -2.754, p = 0.006; Fig. 1). The order of the treatments had no significant effect (LRT: all p > 0.078).

With the exception of the "medium vs. large" treatment, the males' standard length did not significantly correlate with the time spent in front of the stimuli (Pearson and Spearman correlations: all p > 0.177). The males' standard length correlated significantly negative with the time spent in front of the large female stimulus in the "medium vs. large" treatment (Spearman correlation: n = 24, r = -0.536, p = 0.007).

The preference for the larger female tended to be stronger in the "small vs. large" treatment compared to the "small vs. medium" treatment (Wilcoxon test: n = 19, z = -1.913, p < 0.056), as well as in the "small vs. large" compared to the "medium vs. large" treatment (Wilcoxon test: n = 21, z = -1.899, p = 0.058). No significant difference was found between the "small vs. medium" and "medium vs. large" treatment (paired t-test: n = 19, t = 0.894, p = 0.383).

#### Female mate choice

**Figure 2:**
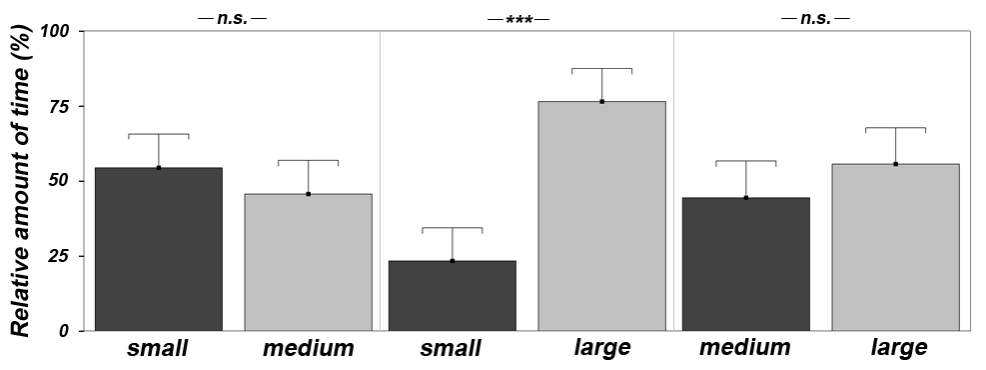
**Female preference**. Female preference expressed as percent of time spent in the association zone in front of male stimuli of different body size. Plotted are means + SD. *** p < 0.001, ** p < 0.01, * p < 0.05, n.s. = not significant.

Females associated high significantly longer with the larger male in the "small vs. large" treatment (paired t-test: n = 23, t = -5.042, p = 0.001; Fig. 2) but showed no significant preferences for the larger one in the two remaining treatments (paired t-tests: small vs. medium: n = 21, t = 0.812, p = 0.427; medium vs. large: n = 24, t = -0.944, p = 0.355; Fig. 2). However, a repeated measures analysis of variance revealed that females high significantly discriminated between the smaller or larger male stimulus (ANOVA: F_1,38 _= 13.910, p = 0.001). The order of the treatments had no significant effect (LRT: all p > 0.148). The females' standard length did not significantly correlate with the time spent in front of the stimuli (Pearson and Spearman correlations: all p > 0.138).

The preference for the larger male was significantly stronger in the "small vs. large" than in both the "small vs. medium" treatment (Wilcoxon test: n = 21, z = -2.950, p < 0.003) and the "medium vs. large" treatment (Wilcoxon test: n = 23, z = -2.516, p < 0.012). No significant difference was found between the "small vs. medium" and "medium vs. large" treatments (paired t-test: n = 21, t = -0.980, p = 0.339).

#### Comparison of preferences between the sexes

In all treatments, males spent significantly relative more time than females with the larger stimulus of the opposite sex, whereas females spent significantly more absolute time in association zones than males (Table 1).

**Table 1:** Differences in preferences between males and females

**Treatment**	**mean ± SD or median (quartiles)**	**t or z**	**n**	**p**
**a)**				
small vs. medium	Males: 78.27% ± 19.50%	4.568	Males: 19	<0.001
	Females: 45.59% ± 24.91%		Females: 21	
small vs. large	Males: 100% (96.38%; 100%)	-2.752	Males: 21	0.006
	Females: 86.59% (50.00%; 100%)		Females: 23	
medium vs. large	Males: 84.56% (51.35%; 100%)	-2.478	Males: 24	0.013
	Females: 50.00% (37.95%; 84.05%)		Females: 24	
**b)**				
small vs. medium	Males: 60.00 s ± 25.00 s	4.427	Males: 19	<0.001
	Females: 93.52 s ± 22.89 s		Females: 21	
small vs. large	Males: 77.48 s ± 34.79 s	-2.377	Males: 21	0.024
	Females: 97.41 s ± 17.10 s		Females: 23	
medium vs. large	Males: 66.75 s (51.00 s; 58.63 s)	-3.177	Males: 24	0.001
	Females:104.25 s (88.50 s; 113.38 s)		Females: 24	

Differences between males and females concerning a) the relative amount of time at the larger stimulus side and b) the absolute time in both association zones. Differences between the sexes were tested with Wilcoxon or t-tests. t or z = test statistics; n = sample size; p = probability.

#### Control experiment

Both sexes significantly preferred the large stimulus (males: mean ± SD = 78.41% ± 21.39%; females: mean ± SD = 79.57% ± 26.54%) over the small stimulus (males: mean ± SD = 21.59% ± 21.39%; females: mean ± SD = 20.43% ± 26.54%) when the extent of nuptial coloration was kept constant between both stimuli (males: paired t-test: n = 11, t = 4.404, p = 0.001; females: paired t-test: n = 13, t = 4.018, p = 0.002).

### Mating and brood care experiment

**Figure 3:**
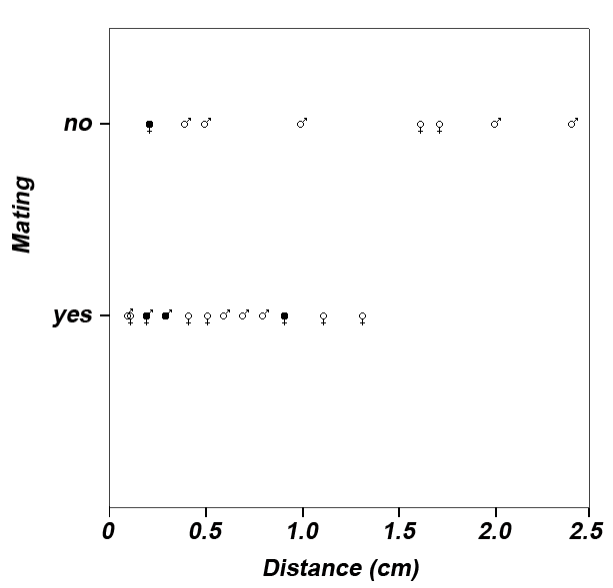
**Likelihood to mate**. The likelihood to mate in *P. taeniatus *was significantly explained by the relative body size distance of pairs (see equation 1 and Results section for statistics). An equal distance was measured in four pairs (filled gender symbols), thus 20 instead of 24 data points are shown. Gender symbols reveal whether the male (male symbol) or the female (female symbol) was relatively larger in a pair. In one case an equal distance between pairs with a relatively larger male and a relatively larger female was measured (male and female symbol combined).

Overall, 15 pairs mated and nine pairs did not mate. The likelihood to mate was significantly explained by the distance of pairs (LRT: χ^2 ^= -4.508, df = 1, p = 0.034; Fig. 3) and the relative size difference of pairs (LRT: χ^2 ^= -4.344, df = 1, p = 0.037). Within the group of unmated pairs, females smaller than the mean female standard length of the population showed significantly more courtship behavior towards the males than females larger than the mean female standard length (Mann-Whitney U test: N_male smaller _= 4, N_male larger _= 5, z = -2.205, p = 0.032). The number of days until spawning was not significantly explained by the females' standard length (Cox regression: LRT_1,24 _= 0.83, beta = 0.545 ± 0.571, p = 0.358). Body size did not significantly correlate with different aspects of parental investment during brood care (e.g. the time males guarded caves, the female cared for eggs, the frequency of warning signals, time near the young: all p > 0.313). The standard length of the females tended to explain the number of eggs (LRT: F_1,12 _= 4.403, p = 0.057) and significantly explained the number of surviving offspring (LRT: F_1,12 _= 6.810, p = 0.023).

## Discussion and conclusion

Males, as well as females of *P. taeniatus *showed preference for larger mates. However, the likelihood to mate in *P. taeniatus *was higher, when the size difference between potential mates was low, thus resulting in size-assortative mating. This corresponds to previous findings in cichlids concerning assortative mating patterns [21,28-31] and preference for larger mates [9,32-34].

The results allow the discussion of the underlying mechanism of the assortment because both individual preferences for body size and their consequences on the likelihood to mate within the same species were investigated. The hypothesis of assortment as a consequence of individual preference for size-assortative mating can be ruled out because no significant positive relationships and one negative correlation between individual body size and preference for the larger stimulus were observed in both sexes. Thus, when given free choice, both sexes of *P. taeniatus *show preference for large potential mates. Furthermore, intra-sexual competition as a mechanism was excluded by the experimental design, although under natural conditions it might also play a role. Due to the mutual character of courtship in the mating system, both males and females are able to accept or reject mates, depending on their individual size compared to that of the mating partner. Consequently, large males could reject small females and *vice versa*. The rejection of relatively small mates is supported by our data since large males did not mate with small females, although these females showed intense courtship behavior. Rejected individuals may fail to mate at all, or have a lower threshold to accept mates that are almost as small as themselves [4]. Thus, the underlying mechanism of size-assortment in *P. taeniatus *is the resolution of sexual conflict over large mates by mutual mate choice [3,4].

Whereas males preferred the larger female in all treatments, females selected the larger male only when the size difference between both stimuli was maximal. Females may not have been able to perceive small differences between stimuli due to the set-up, given that 2D-stimuli may look similar in size when the female is not in the middle of the tank. However, reference objects had been installed. Moreover, males were able to discriminate between the smaller and the larger female stimulus in all treatments, although female stimuli were on average smaller than male models.

Females had a less strong preference for larger mates than males, but spent more time in the association zones. On the one hand this could mean, that females may be less selective for the trait and must present themselves to the choosier sex [1]. On the other hand, each cave-occupying male may initially be attractive for females [35], but an increase of size difference among males may enhance the importance of male body size for female mate choice. A greater difference between stimuli led to a significant preference for the larger male stimulus, indeed. Thus, females may be choosy, but a smaller difference in male size may not be important. As paternal investment of *P. taeniatus *in brood care seems to be independent of males' body size in our study, the offspring may gain the same investment from a medium-sized or large male. However, other possible advantages of larger males, e.g. protection against predators, were not investigated in this study.

The adaptive significance of sexual selection on body size has been discussed in many studies [14,36]. In the present study, female body-size in *P. taeniatus *indicated the number of surviving offspring, thus supporting previous findings that females' body size reveals fecundity [e.g. [21] and citations therein, [37]]. Thus, preference for large females could increase male fitness. The impact of large male body size was not investigated in the present study. Larger males may be more successful in intra-sexual competition, offering better territories or protection against predators [9,38,39]. Furthermore, if larger males in *P. taeniatus *have advantages in intra-sexual competition (TT, unpublished data) like larger males in other species [e.g. [40-42]] females may prevent the loss of a brood when preferring the larger male.

The parental investment of males – as well as females – seemed to be independent of body size during brood care in *P. taeniatus*. On the one hand this could mean, that body size does not signal the quality of a potential mating partner concerning brood care. On the other hand, brood care may be performed by each sex on a constant high level independent of its own or the partner's body size. However, in our study parental investment of *P. taeniatus *was measured in size-assortative mated pairs which do not represent the largest individuals of the population used. Of course, parental investment was assessed under optimal laboratory conditions leaving an influence of body size under more stressful conditions open.

There are two general problems when investigating the effects of body size on mating decisions. First, in many species body size may be correlated to other traits. Thus, it is impossible to disentangle which trait is ultimately selected for. Second, the relative proportion of other traits with respect to body size may be more important, making body size relevant as a combined trait [e.g. [32]]. The control experiment in which the area of nuptial coloration was kept constant independent of body size revealed a strong preference for the larger stimulus in both sexes, underlining that body size is decisive for each sex independent of the extent of the nuptially colored area. We cannot exclude that there are unknown sexual traits that shift with the downscaling of the stimulus' body size. Nonetheless, this method of investigating body size provides the highest possible degree of standardization.

In general, the sex with the higher reproductive costs is expected to be more selective which is usually assumed to be the female [see [43] for a recent model explaining assortment by female mate choice]. Our results show that *P. taeniatus *may be a good model species for research questions concerning mutual mate choice. A recent review revealed that mutual mate choice plays a role in many species [see [44] for an overview of species showing mutual mate choice or positive assortment]. Thus, assortment by mutual mate choice may be an important mechanism for the resolution of sexual conflict over quality traits in many species.

## Methods

### Experimental animals

All individuals of *P. taeniatus *were bred and maintained under standardized laboratory conditions [see [22]]. The parents of the test subjects originated from the river Moliwe in Cameroon (04°04'N/09°16'E), West Africa. Individuals were F1- and F2-offspring from 23 outbreeding pairs and were raised in mixed-sex family tanks (80 × 30 × 30 cm). The tanks were surrounded with opaque plastic sheets to avoid visual contact to other aquaria. Test fish were 1–2 years old and reproductively active. The water temperature was kept at 25 ± 1°C and natural light conditions were given (L/D 12/12). Nutrition was provided once a day with a mixture of frozen chironomid larvae and *Artemia *ssp.

### Mate choice using computer animations

#### Preparation of artificial stimuli

We took digital photographs (Olympus Camedia Widezoom 5060) of a nuptially colored male and female to obtain source data for two-dimensional fish models. 2D-models are a sufficient method to test mating preferences in *P. taeniatus *for certain traits like body size (SAB, HK, TT, TCMB unpublished data). The pictures were saved in RAW-format to avoid the loss of coloration data due to algorithmic compression. They were white-balanced during import to Adobe Photoshop CS2. The size dimensions of the pictures were manipulated for the three treatments (Table 2).

**Table 2:** Total lengths of the manipulated images

**Treatment**	**Female stimuli size (cm)**	**Male stimuli size (cm)**
small vs. medium	3.5 vs. 4.5	5.0 vs. 6.5
small vs. large	3.5 vs. 5.5	5.0 vs. 8.0
medium vs. large	4.5 vs. 5.5.	6.5 vs. 8.0

Overview of pairs of artificial stimuli used in measuring mating preferences for body size. Each test fish conducted all three treatments in random order.

To achieve moving animations of the models, we used "The GIMP 2.20 with animation package". A grey background image (1024 × 400 px) was created (RGB: 238,238,238) including a reference object for each sex (plant for test males/breeding cave for test females, in the middle of the image). Each animation consisted of 30 frames per second which is an established method to present artificial stimuli to test fish [26,27,45]. Each stimulus moved a horizontal pathway from one side of the monitor to the other for a period of 15 seconds, including a two second stop in the middle. After that, it recurred horizontally and moved back in the same time frame.

For each sex we created three different experimental treatments (Table 2) with different body size of the stimuli. The body size of each stimulus was adapted for a monitor and video resolution of 1024 pixel width.

#### Experimental design

Experiments were conducted between 01 June 2007 and 02 August 2007. Before the start of the trials 30 males and 24 females, that varied greatly in body size (Table 3), were randomly chosen and individually isolated in separate tanks (25 × 15 × 15 cm) for a minimum period of two days. The mating readiness of each test fish was determined visually on the basis of the ventral coloration, as well as the display of courtship behavior in the family tanks [22,24]. The isolation tanks were surrounded at the broad sides by print-outs of the animation's background image and opaque, grey partitions at the longer sides, thus ensuring that fish did not interact with other isolated individuals and were able to habituate to the background and reference objects also used in the trials. Java moss, *Vesicularia dubyana*, was added to provide shelter for the females, whereas the male tanks were equipped with a breeding cave. All other conditions were similar to those of the mixed-sex tanks. Each individual conducted the three experimental treatments in random order. Test fish were transported to the experimental set-up in their isolation tank, thus reducing stress by leaving the fish in its familiar habitat.

**Table 3:** Standard lengths (minimum-maximum, mean ± SD) of test fish for each treatment

**Treatment**	**Female size (cm)**	**Male size (cm)**
small vs. medium	3.3 – 4.7, 3.85 ± 0.39	4.8 – 6.5, 5.34 ± 0.51
small vs. large	3.3 – 4.7, 3.85 ± 0.41	4.8 – 6.8, 5.50 ± 0.58
medium vs. large	3.3 – 4.7, 3.85 ± 0.40	4.8 – 6.8, 5.48 ± 0.55

The set-up was illuminated by a fluorescent tube (37W) installed one meter above the middle of the tank. Additionally, white Styrofoam surrounded the tank. Tanks containing the test fish were placed between two CRT monitors of the same model (EIZO Flex Scan F520, 85 Hz, connected to a Matrox G550 PCIe graphic board). The level of the bottom of the tank was justified so that it coincided with the lower margins of the monitor screens. An association zone of 5 cm in front of each monitor was marked on the white Styrofoam under the tank creating a 15 cm neutral zone in between.

It was determined randomly which stimulus was presented on which monitor. During an acclimatization period of 15 minutes both screens showed the grey background. After acclimatisation, the stimuli appeared simultaneously on both monitors [46]. In which direction the stimuli were moving (animations) was randomly determined. We recorded two minutes after the fish had visited the first association zone, which is a time frame established in recent studies [47-49]. After recording, the empty background was shown again for five minutes. The trial was then repeated with switched monitor sides of the stimuli.

After the three experimental treatments, the standard length and mass of the test fish was measured. A naïve observer analyzed the video recordings. Mating preferences were measured as association time near a stimulus of the opposite sex, which reliably predicts mating decisions in both male and female *P. taeniatus *[[22] and references therein, [24], TT, TCMB, N. Henning, HK unplubished data]. The time spent in each association zone was calculated over a period of two minutes after the fish had first visited an association zone. In the analyses, relative proportion of time spent in one of the association zones was used. For each test fish, we averaged the time spent in front of each stimulus in the first and the second trial, thus excluding possible side biases. In each treatment a different number of test fish completed both trials, thus sample sizes varied among treatments. To compare the preferences for stimuli between the three treatments, we subtracted the relative time spent in front of the smaller stimulus from the larger stimulus for each sex.

#### Control experiment

Small stimuli in the computer animations were down-sized versions of the larger stimuli. Thus, other sexual traits possibly playing a role in sexual selection were also down-scaled like the extent of nuptial coloration of each sex. We therefore carried out a control experiment to test whether preference for body size in each sex was independent from the extent of nuptial coloration. Experiments were conducted between 03 December 2007 and 14 December 2007. The methods complied with the set-up described above. We used the small and the large stimulus for each sex from the former experiments as stimuli, but kept the area of nuptial coloration constant in both stimuli. Both stimuli showed the extent of nuptial coloration of the small stimulus.

### Mating and brood care experiment

Observations were made in winter 2007. We formed 24 outbreeding pairs differing in relative body size distance by taking nuptially colored individuals of each sex from family tanks, measuring their standard length, and sorting males (range: 4.9–7.5 cm) in descending and females (range: 3.4–5.0 cm) in ascending order of body size. Then the largest male was combined to the smallest female and *vice versa *(i.e. largest relative size distance, see equation 1 and 2 below) with a continuous shift of relative size distance of pairs in between. The pairs were allocated randomly to aquaria (30 × 40 × 43 cm), which were filled with 2/3 parts of tap water and 1/3 parts of osmotic water and tempered at 25 ± 1°C. Two observers (SHS, SAB) recorded behavioral patterns of each pair for 10 min daily over a period of eight weeks. The observation order was random and determined daily. Unmated pairs were stimulated weekly by renewing 1/3 of the water volume with the 2:1 tap/osmotic water mix, but observations were stopped for pairs that were still unmated after three weeks.

After spawning, the cave was shortly removed and the number of eggs was counted. The egg number of one female could not be determined. We recorded several behavioral patterns during courtship and parental care [see [22] for details]. We recorded every 30 seconds whether the male guarded the cave or not, or whether the female cared for the eggs. Mating was counted as successful when fry left the breeding cave and the parents showed brood care for the free swimming offspring. The number of surviving young was counted four weeks after the fry had left the breeding cave. The sample size here was reduced by one, as one pair cannibalized their fry after three weeks. Fry were fed with living *Artemia nauplii*, and when they grew older with a mix of frozen *Artemia*, chironomid, and mosquito larvae.

In order to analyze the likelihood to mate we calculated the body size distance of each pair relative to the mean population size difference, called "distance of pairs" from now on (Equation 1). However, males and females achieve different maximum body sizes: a large female and a large male have different absolute body sizes, but may be the same in relative size compared to other individuals of their own sex. Thus, we additionally calculated the relative size difference between a male and a female in relation to the mean body size of each sex in the population, so called "relative size difference of pairs" from now on (Equation 2).

(1)

(2)

### Statistics

Parametric statistics were used when data did not significantly deviate from normal distribution according to Kolmogorov-Smirnov tests with Lilliefors correction. Given test probabilities are two-tailed throughout. p-values <0.05 were considered statistically significant. Female mate preferences in the computer animation experiments were additionally analyzed by a repeated measures analysis of variance. Because male data were not normally distributed (not even after transformation), only non-parametric test statistics are shown. The likelihood to mate in relation to the distance of pairs (see equation 1) and relative size difference of pairs (see equation 2) were analyzed with generalised linear models ("glm"), with binomial error distribution and logit link function. The impact of body size on the number of eggs or surviving young was analyzed with linear models ("lm"). Likelihood-ratio tests (LRT) assessed whether the removal of a variable caused significant decrease in model fit. Reported p-values of models refer to the increase in deviance when the respective variable was removed ("lm": F-statistics; "glm": Pearson's chi-square). Analyses were performed using SPSS 12.0, and LRTs were calculated using R 2.6.1 statistical package.

## Authors' contributions

SAB participated in the design of the study, collected data, performed statistical analyses and wrote the paper. HK participated in the design of the study, the statistical analyses and the writing of the paper. SHS collected data and performed statistical analyses. TT participated in the design of the study, statistical analysis and the writing of the paper. TCMB participated in the design of the study and the writing of the paper. All authors read and approved the final manuscript.
